# Cadmium burden and the risk and phenotype of prostate cancer

**DOI:** 10.1186/1471-2407-9-429

**Published:** 2009-12-10

**Authors:** Yi-Chun Chen, Yeong S Pu, Hsi-Chin Wu, Tony T Wu, Ming Kuen Lai, Chun Y Yang, Fung-Chang Sung

**Affiliations:** 1 Department of Health Management, I-Shou University, Kaohsiung 824, Taiwan; 2 Institute of Environmental Health, National Taiwan University, Taipei 100, Taiwan; 3 Department of Urology, National Taiwan University Hospital, Taipei 100, Taiwan; 4 Division of Urology and Institute of Environmental Health, China Medical University and Hospital, Taichung 404, Taiwan; 5 Division of Urology, Kaohsiung Veterans General Hospital, Kaohsiung 813, Taiwan; 6 Department of Public Health, Kaohsiung Medical University, Kaohsiung 807, Taiwan

## Abstract

**Background:**

Studies on the association between prostate cancer and cadmium exposure have yielded conflicting results. This study explored cadmium burden on the risk and phenotype of prostate cancer in men with no evident environmental exposure.

**Methods:**

Hospital-based 261 prostate cancer cases and 267 controls with benign diseases were recruited from four hospitals in Taiwan. Demographic, dietary and lifestyle data were collected by standardized questionnaires. Blood cadmium (BCd) and creatinine-adjusted urine cadmium (CAUCd) levels were measured for each participant. Statistical analyses measured the prostate cancer risk associated with BCd and CAUCd separately, controlling for age, smoking and institution. BCd and CAUCd levels within cases were compared in relation to the disease stage and the Gleason score.

**Results:**

High family income, low beef intake, low dairy product consumption and positive family history were independently associated with the prostate carcinogenesis. There was no difference in BCd levels between cases and controls (median, 0.88 versus 0.87 μg/l, p = 0.45). Cases had lower CAUCd levels than controls (median, 0.94 versus 1.40 μg/g creatinine, p = 0.001). However, cases with higher BCd and CAUCd levels tended to be at more advanced stages and to have higher Gleason scores. The prostate cancer cases with Gleason scores of ≥ 8 had an odds ratio of 2.89 (95% confidence interval 1.25-6.70), compared with patients with scores of 2-6.

**Conclusion:**

Higher CAUCd and BCd levels may be associated with advanced cancer phenotypes, but there was only a tenuous association between cadmium and prostate cancer.

## Background

The incidence and mortality rates of prostate cancer vary markedly among ethnic groups, being low in Asian and much higher in Western populations. Nonetheless, the incidence of the disease in Taiwan increased more than 3-fold during the 1990s [1]. In addition to factors such as age, race and inherited predisposition, dietary factors, particularly fat, may also play important roles in prostate carcinogenesis [2,3]. Various other environmental and occupational exposures have also been shown to be associated with prostate cancer. These include cadmium exposure, which has been investigated but with inconclusive results. The association of cadmium exposure with prostate cancer was reported by Potts in 1965 that three cadmium battery workers had died of the disease [4]. Several subsequent occupational and geographical studies gave conflicting results [5-11].

A geographical study in Spain showed that an area with elevated cadmium contamination also had a high population incidence of prostate cancer. However, the authors did not conclude that cadmium was the determining cause of prostate cancer; which probably has more to do with nature occurrence of the chemical than with human activity [9]. Occupational studies on cadmium exposure may be not general population representative because workers suffer much greater exposure than the general population. Similarly, ecological studies have been unable to address relevant confounding factors adequately. In these occupational and ecologic studies, no cadmium was measured in vivo to reflect the real human exposure and this may have led to conflicting results. To determine whether cadmium is an etiological or only a secondary factor in prostate carcinogenesis, measurement of cadmium levels in vivo may be helpful. Feustel et al. [12] measured the cadmium contents of prostate tissue and found elevated levels in malignant tissues. Vinceti et al. found that men in Italy with higher toenail cadmium are at higher risk for prostate cancer [13]. No study has investigated the cadmium burden using blood cadmium (BCd) and urine cadmium (UCd) levels among men with no occupational exposure. This study determined and compared the BCd and creatinine-adjusted urine cadmium (CAUCd) levels among prostate cancer patients with different disease phenotypes and in controls.

## Methods

### Subjects

Cases, which were histologically confirmed prostate adenocarcinoma, were recruited from four medical centers: two hospitals from northern, one from central and one from southern Taiwan. This recruitment plan was adopted in an attempt to represent the likely span of cadmium exposure across Taiwan as a whole. Controls were age-matched male patients also from the same four hospitals who had received medical attention from the services of urology, family medicine, orthopedics, ophthalmology, otolaryngology and other services. Subjects acquired from the urology service were limited to those with inguinal hernia or urolithiasis. Subjects with other malignancies, hormonal disorders or benign prostatic diseases and men with the prostate specific antigen >= 4 ng/ml were excluded from the control group. This study was performed with the approval of the ethics committee.

### Data collection

With physician approval, all participants recruited gave their informed consent prior to the study interview. Trained interviewers conducted personal interviews with all participants during hospital visits using standard questionnaires. The questionnaires covered information about socio-demographic characteristics, occupation history, tobacco and alcohol use, diet, physical activity, height and weight history, medical history, and family history of prostate cancer.

Using a food frequency questionnaire, both cases and controls reported dietary intake history from 10 years ago, determined by personal recall. The questionnaire was a validated instrument and had been used in previous studies [14,15]. It had been modified during the past few years to adapt it to the changing environment in our society. The questionnaire contained the most frequently consumed food items among Chinese, including poultry, pork, beef, egg, fish, seafood, diary products, vegetables and fruits, tofu, herbal preparations as food supplement, and eating out including banquets. Food consumption was reported at daily, weekly or monthly frequency. The questionnaire also asked information about smoking and some drink items including alcohol, coffee, soybean milk, tea, etc. Since the dietary history was determined using the semi-quantitative food frequency questionnaire, this version was validated with a Cronbach's alpha ranging from 0.75 to 0.84.

Cancer stages and Gleason scores were extracted from medical records. Among the 261 cases, these data were available, respectively, for 220 (84.3%) and 210 (80.5%) cases. Cancer stages were recoded in Taiwan by the American Urology Association (AUA) System, and Tumor, Node and Metastasis (TNM) staging [1]. In this study, the cancer stage recorded by TNM staging system was transformed to the AUA system by a Urology specialist. Pathologists evaluated the architectural pattern of the glands of prostate tumors on the basis of histological examinations and gave scores representing the phenotype of prostate cancer. The score ranges from 2 to 10, with 10 being associated with the worst phenotype or prognosis.

### Specimen collection and cadmium analysis

Since cadmium has a biological half-life of decades in humans, blood and urine levels are considered fairly stable throughout the day. Therefore, single spot urine and blood samples were collected during scheduled appointments instead of at a standardized time during the day. The urine sample was collected in a sterile plastic cup and an aliquot of 10 ml was transferred to a conical-bottomed cadmium-free plastic tube. Blood specimens of 2-3 ml were collected in pre-screened heparin tubes, also from lots verified to be free of cadmium contamination. Both urine and blood specimens were stored at 4°C if analyzed within a week or at -20°C if they were to be analyzed a week or more later. One ml of urine was used for creatinine determination in order to normalize the urine cadmium levels.

One hundred μl of blood plus 900 μl of matrix modifier (0.2% nitric acid, 0.5% Triton^®^-X-100 and 0.2% (NH_4_)_2_HPO_4_) or 100 μl of urine plus 200 μl of matrix modifier (25% NH_4_H_2_PO_4 _and 1.25% Mg(NO_3_)_2_) were prepared for analyses [12]. Specimens, quality control samples, and standards admixed with matrix modifier were analyzed for cadmium contents using a Perkin-Elmer Model 5100 PC atomic absorption spectrometer with Zeeman background correction. For quality control, reference blood samples (NYCOMED PHARMA AS, Oslo, Norway) were used. Urinary cadmium analysis was verified every other month using the Inter-Laboratory Comparison Program, Le Centre de Toxicology du Québec (Sainte-Foy, QC, Canada). The recovery rates for spike tests were 100.7 ± 3.5% for urine analysis and 100.9 ± 3.6% for blood analysis. The urine cadmium levels were adjusted for urinary creatinine content.

### Statistical analyses

Contingency tables with Chi-square tests were used to examine the categorical variables to compare cases and controls. The associations with major exposures and covariates were examined. We selected several variables from three major categories - sociodemographics, lifestyle, and food consumption - as the covariates. BCd and CAUCd levels were examined by both parametric and non-parametric methods. Since neither level was normally distributed, medians BCd and CAUCd levels were compared between cases and controls using the Wilcoxon rank-sum test. Multivariate logistic regression models were used to estimate the contribution of other factors such as socioeconomic status, dietary habit and family history. We also compared the BCd and CAUCd levels by Kruskal-Wallis test with post hoc comparisons (Dunn test) in cases grouped according to tumor stages (A+B, C, and D) and Gleason scores (2-6, 7, and >= 8) to check whether cadmium levels differed among graded malignant phenotypes. The analyses that estimated the odds ratios for association between cancer grade, by stage, and the Gleason score, and median levels of CAUCd (<= 1.12 vs. > 1.12 μg/g creatinine) and BCd (<= 0.87 vs. > 0.87 μg/L) were based on available samples from cases. The significance level was taken as p < 0.05 in this study.

## Results

A total of 261 cases (age: 72.1 ± 6.8 years) and 267 controls (age: 71.3 ± 7.2 years) were recruited. Comparisons of sociodemographics, lifestyle and food consumption showed no significant differences between cases and controls in body mass index, education, occupation, smoking, drinking or regular exercise. However, cases had a significantly higher family income (p = 0.001). Subjects with family histories of prostate cancer had a 3.4-fold greater risk than those without such histories (p = 0.02). Interestingly, cases tended to consume less beef (p = 0.04) and dairy products (p = 0.046) but ate out more often (p = 0.02) than controls (data not shown).

Among the participants, 234 (89.7%) cases and 248 (92.9%) controls provided urine samples and 176 (67.4%) cases and 147 (55.1%) controls donated blood samples for cadmium measurements. Cases and controls who provided urine specimens were similar in mean ages; but among those who donated blood samples, the mean age was 1.8 years greater in cases than in controls (data not shown). Cases who provided either urine or blood specimens were wealthier and ate out frequently. There was no significant difference in education between cases and controls.

**Figure 1:**
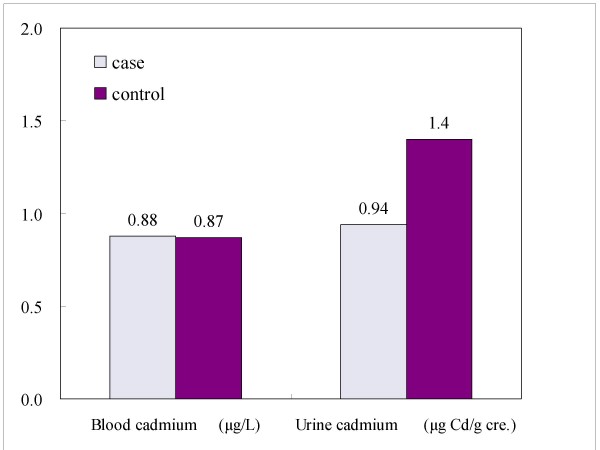
**Comparisons of median blood and urine cadmium levels between prostate cancer cases and controls**.

Figure 1 shows that there was no significant difference between cases and controls in median BCd levels (0.88 and 0.87 μg/l, respectively). The median CAUCd levels were significantly lower in cases than in controls (0.94 and 1.40 μg/g creatinine, respectively, p = 0.001 by Wilcoxon rank-sum test).

Table 1 shows that after adjusting for age, smoking and the medical institution from which the subjects were recruited, high family income, low beef intake and low dairy product consumption remained significant independent factors predicting an elevated risk of prostate cancer when BCd was included in the model. In addition to these three factors, CAUCd and family history were also independent factors associated with the risk of prostate cancer when CAUCd was introduced into the model.

**Table 1:** Multiple logistic regression analyses for factors associated with prostate cancer *

Variable	Odds ratio (95% CI)	*p*	Variables	Odds ratio (95% CI)	*p*
Blood cadmium, μg/l			Urine cadmium, μg Cd/g creatinine		
≤ 0.87	1.0	0.24	≤ 1.12	1.0	0.003
> 0.87	1.44 (0.78-2.64)		> 1.12	0.49 (0.31-0.78)	
Family income			Family income		
Median/low	1.0	0.002	Median/low	1.0	0.002
High	2.86 (1.45-5.61)		High	2.25 (1.35-3.73)	
Beef			Beef		
Less	1.0	0.041	Less	1.0	0.033
Frequent	0.53 (0.29-0.98)		Frequent	0.60 (0.37-0.96)	
Dairy products			Dairy products		
Less	1.0	0.065	Less	1.0	0.056
Frequent	0.49 (0.23-1.04)		Frequent	0.58 (0.33-1.01)	
Eating out			Eating out		
Less	1.0	0.309	Less	1.0	0.049
Frequent	1.39 (0.74-2.61)		Frequent	1.62 (1.0-2.6)	
Family history		0.095	Family history		0.045
No	1.0		No	1.0	
Yes	4.41 (0.77-25.1)		Yes	3.47 (1.03-11.7)	

* Analysis was done by controlling for subjects' ages, smoking status and medical institutions; CI = confidence interval.

The distributions of tumor stages and Gleason scores were comparable among all four hospitals. The median BCd and CAUCd levels of cases with different tumor stages and Gleason scores are shown in Table 2. Both BCd and CAUCd levels tended to increase as tumor stage or Gleason score progressed. By the non-parametric Kruskal-Wallis test, significant differences in both BCd and CAUCd levels were seen among patients with different Gleason scores (p = 0.013 and 0.03, respectively). There was a significant difference in CAUCd levels (but not BCd levels) among patients at different stages (p = 0.043). Post hoc comparison showed that patients with a Gleason score of 7 had a higher BCd level than those with a score of 6 or less (p = 0.004). Similarly, patients with a Gleason score of 8 or more had a higher CAUCd level than those with a score of 6 or less (p = 0.03). Patients with stage D disease had a higher CAUCd level than those with either stage A+B (p = 0.04) or C (p = 0.04). The data were also log-transformed and compared among stage groups and among Gleason score groups using ANOVA. Similar significance results were obtained.

**Table 2:** Median and 5-95th percentile range of blood and urine cadmium levels in prostate cancer patients by stage and Gleason score

Stage	N	Median (5-95%)	p*	Gleason score	N	Median (5-95%)	p*
**Blood cadmium (μg/l)**							
A+B	56	0.85 (0.12-3.52)	0.285	2-6	88	0.74 (0.12-2.39)	0.013
C	34	0.89 (0.11-2.62)		7	41	1.03 (0.40-4.45)	
D	73	0.92 (0.24-2.95)		≥ 8	22	0.91 (0.24-2.89)	
							
**Urine cadmium (μg Cd/g creatinine)**							
A+B	72	0.76 (0.10-3.95)	0.043	2-6	107	0.72 (0.12-3.72)	0.030
C	43	0.74 (0.14-2.91)		7	54	0.96 (0.13-4.37)	
D	84	1.12 (0.21-4.37)		≥ 8	30	1.48 (0.17-7.36)	

* By Kruskal-Wallis test

**Figure 2:**
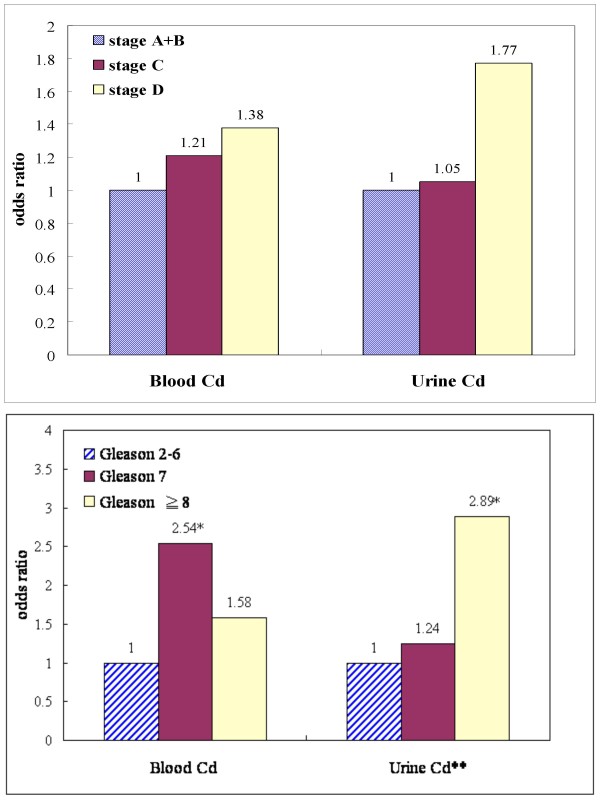
Odds ratio (OR) by stage (C, D vs A+B) and Gleason score (≥ 7 vs. 2-6) of association of prostate cancer with cadmium levels in urine and blood^§ ^(*p < 0.05; **p for trend = 0.04). ^§ ^Using the median level of cases as the cutoff value for risk determinations, 0.87 μg/l for blood cadmium and 1.12 μg/g creatinine for urine cadmium.

Further data analyses showed that prostate cancer patients diagnosed at the advanced stage had greater odds ratios (OR) of having higher CAUCd and BCd, but this was not significant (Figure 2). Compared with patients with the Gleason scores of 2-6, the OR of prostate cancer being associated with CAUCd levels higher than 1.12 μg Cd/g creatinine increased to 1.24 (95% CI 0.64-2.42) for those with a score of 7, and to 2.89 (95% CI 1.25-6.70) for those with a score of 8 (p for trend 0.04). Higher Gleason scores were also associated with higher BCd.

## Discussion

Population aging, lifestyle changes and increased screening have resulted in a rapid increase in both the incidence of and mortality from prostate cancer in low incidence Asian countries. In our previous study [14,15] and the present study, we investigated the association between prostate cancer and socio-demographics, life style and dietary factors. In this study, we also investigated the association between the cadmium burden and prostate cancer risk, which has not previously been clearly delineated for men based on the measured BCD and CAUCd.

Animal and occupational studies have strongly suggested that cadmium is carcinogenic to the prostate [5,6,8,16-19]. Waalkes et al. demonstrated a dose-response relationship in a rodent model when tumors were induced by injecting cadmium subcutaneously [19]. Animal experiments are usually conducted at the maximum tolerated doses or supra-normal levels of a certain toxin. Very few studies have focused on general human populations [13,20,21].

A small-scale Nigerian epidemiological study showed that the plasma cadmium concentration is significantly higher in subjects with prostate cancer than in normal subjects or patients with benign prostatic hypertrophy [21]. There were only 12 cancer patients in that study all with locally advanced or metastatic disease. Vinceti et al. used toenail cadmium as an indicator of body burden and found a significant relationship between cadmium and prostate cancer; but, the statistical methods used were unstable, again because of the small sample size [13]. Another study using urinary cadmium as indicator showed a weak positive relationship between cadmium and prostate-specific antigen [20].

Occupational studies have also shown some evidences of elevated risks upon substantial exposure [5,6,8]. BCd levels normally range from 0.4 to 1.0 μg/l for nonsmokers and from 1.4 to 4.0 μg/l for smokers [22]. In the event of environmental or occupational exposure, BCd levels up to 10 μg/L may lead to renal dysfunction and/or osteopenia [23]. CAUCd levels normally average 0.35 and > 2 μg/g creatinine for nonsmokers and smokers, respectively [24]. For individuals with substantial occupational exposure, BCd may rise up to 50 μg/L, and CAUCd up to 50 μg/g creatinine [25]. This suggests that a very high body burden of cadmium can occur in contaminated working environments, where the cadmium exposure is usually heavier than that incurred by smoking. However, it may not be appropriate to extrapolate data from rodent and occupational studies, in which high-dose exposures infer a relationship between cadmium burden and carcinogenesis in the general human population. This may explain why general observational studies, where no evident environmental exposure is present, have failed to reveal consistent results about the association of cadmium.

The present study demonstrated that men with prostate cancer have lower CAUCd levels and there is no significant difference in BCd level between cases and controls. However, patients with higher Gleason scores were more likely have higher cadmium burdens than patients with lower scores.

The primary sources of cadmium exposure for the general population are from smoking and contaminated food [26]. BCd primarily reflects recent exposure rather than whole body burden, while CAUCd is more indicative of long-term body burden with some contribution from recent exposure. Therefore, CAUCd seems a better marker than BCd for assessing the association between cadmium exposure and cancer risks [17,27]. Our data showed no significant difference in BCd levels between cases and controls. To our surprise, subjects with higher CAUCd had a significantly lower risk for prostate cancer. This result contradicted our previous belief that UCd might parallel BCd in the association with the cancer risk. A possible explanation is that cadmium is carcinogenic to the prostate; prostate cancer may retain a significant amount of cadmium (nor related to the circulation levels) [12,28] and allow only trace amounts to be excreted via the kidney, in contrast to a benign prostate that does not "absorb" cadmium and undergo malignant transformation. It may not matter how much cadmium enters the body in total. What matters is how much the prostate "traps" the cadmium and reacts to it. The change in CAUCd that we have observed merely reflects a balance in cadmium content between prostate tissue, blood and urine.

It may take decades for prostate epithelia to initiate and promote during carcinogenesis. It also takes years for the body cadmium burden to be eliminated after exposure. Once absorbed, cadmium is transported through the blood stream to various tissues. Cadmium in the liver and kidney accounts about 50% of the total body burden [26], as these organs synthesize large amounts of metallothionein, a metal-binding protein with a high affinity for cadmium. Cadmium is eliminated very slowly from the body. Its biological half-life of cadmium has been estimated as approximately 25-30 years in humans [29]. This long biological half-life may well set the stage for neoplastic transformation.

Our comparison of cadmium levels among cases with graded malignancies revealed potentially interesting results deserving careful interpretation and further study. In patients with prostate cancer, there was a trend towards higher tumor stages and worsening Gleason scores as the BCd or CAUCd levels increased, except that the association of BCd with tumor stages was not significant. Higher cadmium levels appeared to be associated more with the Gleason score than with tumor stage. CAUCd correlated significantly more strongly than BCd with the malignant phenotype of prostate cancer. The reason for this is unclear. It has been shown that levels of cadmium in prostate tissue are higher in more advanced diseases [12,28]. Patients with more advanced diseases may have first entrapped more cadmium into the prostate during carcinogenesis and then excreted more cadmium into the urine during disease progression and tumor necrosis than those with more localized diseases or low-grade tumors. However, as cadmium remains largely entrapped in the cancerous prostate, the CAUCd levels in cases are still lower than that in controls, especially in cases at earlier stages and with lower grades.

Although we believe that low urinary cadmium levels are associated with the entrapment of cadmium in the cancerous prostate, it is also possible that some people who are more sensitive to cadmium than others and thus may therefore develop cancer even at low exposure levels.

The strength of this study includes the recruitment of study subjects from medical centers distributed across all areas in Taiwan, which may have reduced the bias from patient selection, environmental exposure or dietary differences. This study is one of the few that have actually measured the cadmium burden in the general population in order to study its association with the risk of prostate cancer. Recall bias in terms of multiple items of risk exposure was minimal. Since not all subjects donated specimens for cadmium determination, it is possible that differences in cadmium burden exist between specimen donors and non-donors. However, this bias may also be minimal because the smoking rates and socio-demographic parameters (such as education level) were similar among donors and non-donors. The other strength is that this is the first study to have associated cadmium exposure with the stage and Gleason score of the disease. This interesting relationship between prostate cancer phenotypes and cadmium levels may be due to an effect of specific tumor type and not to an etiological role of the metal.

## Conclusion

This study provides new information regarding the non-occupational cadmium exposure and the development of prostate cancer. The cadmium level might be an indicator of the progress of the disease, greater level in the more advanced stage. Little evidence can be advanced to prove that cadmium is a predictor of prostate cancer. Further studies to clarify the pathways of cadmium metabolism in the prostate and the genetic susceptibility that interacts with cadmium may help in the understanding of cadmium-mediated prostate carcinogenesis.

## List of abbreviations

CAUCd: creatinine-adjusted urinary cadmium level; UCd: Urine cadmium level; BCd: blood cadmium level; OR: odds ratio; CI: confidence interval.

## Competing interests

The authors declare that they have no competing interests.

## Authors' contributions

FS is the PI who initiated the study design, and carried out this study. YPu, HW, TW and ML recruited subjects and conducted diagnosis. YC and YPu carried out laboratory work and initiated manuscript development. CY participated in the study design and data interpretation. All authors participated in the conception and design of the study, and in the writing and approval of the manuscript.

## Pre-publication history

The pre-publication history for this paper can be accessed here:

http://www.biomedcentral.com/1471-2407/9/429/prepub
